# IR Spectroscopy as a Diagnostic Tool in the Recycling Process and Evaluation of Recycled Polymeric Materials

**DOI:** 10.3390/s25196205

**Published:** 2025-10-07

**Authors:** Kaiyue Hu, Luigi Brambilla, Chiara Castiglioni

**Affiliations:** Dipartimento di Chimica, Materiali e Ingegneria Chimica Giulio Natta, Politecnico di Milano, 20133 Milano, Italy; kaiyue.hu@polimi.it (K.H.); luigi.brambilla@polimi.it (L.B.)

**Keywords:** plastic waste, polymers sorting, structural characterization, chemical recognition, polymer morphology, secondary raw materials

## Abstract

Driven by environmental concerns and aligned with the principles of the circular economy, urban plastic waste—including packaging materials, disposable items, non-functional objects, and industrial scrap—is increasingly being collected, recycled, and marketed as a potential substitute for virgin polymers. However, the use of recycled polymers introduces uncertainties that can significantly affect both the durability and the further recyclability of the resulting products. This paper demonstrates how spectroscopic analysis in the mid-infrared (MIR) and near-infrared (NIR) regions can be applied well beyond the basic identification of the main polymeric component, typically performed during the sorting stage of recycling processes. A detailed interpretation of spectral data, based on well-established correlations between spectroscopic response and material structure, enables the classification of recycled polymers according to specific physicochemical properties, such as chemical composition, molecular architecture, and morphology. In this context, infrared spectroscopy not only provides a reliable comparison with the corresponding virgin polymer references but also proves particularly effective in assessing the homogeneity of recycled materials and the reproducibility of their properties—factors not inherently guaranteed due to the variability of input sources. As a case study, we present a robust protocol for determining the polypropylene content in recycled polyethylene samples.

## 1. Introduction

The environmental sustainability of plastic object production can be significantly improved by developing recycling strategies that ensure reliable assessment of the quality and reproducibility of the recycled polymeric material [1,2,3].

Chemical recycling, which involves depolymerizing the polymeric component to obtain small molecules (monomers) that can be reused as raw materials in subsequent polymer synthesis processes, is potentially the most reliable route [4,5]. In principle, it can yield a final product (the polymer) that is identical to the one obtained from conventional sources. However, the complexity and cost of chemical processes have so far made mechanical/thermal recycling the most widely adopted strategy for polymer materials [3,4,5].

Ideally, a thermoplastic polymer can be melted and reformed an infinite number of times at relatively low temperatures, thus with limited energy consumption, without losing its physicochemical properties.

This unique feature makes mechanical/thermal recycling feasible: plastic waste—once sorted by polymer type—is shredded into fragments, which are then melted and extruded to produce pellets ready for market use as secondary raw materials for manufacturing new plastic products. Although this process may appear simple when described schematically, each of its steps has critical aspects that strongly impact the quality of the final product. We can identify (i) issues related to the intrinsic characteristics of the plastic waste and (ii) issues related to the recycling process itself.

Waste material-related issues concern the accuracy of polymer sorting, and the presence of contaminants or foreign fragments that were not removed. These problems are especially common in post-consumer plastic waste, while post-industrial waste sources typically allow for predefined selection of the input polymer type.

It is also essential to recognize that polymers in finished products are always used in formulations. Part of the formulation depends on the raw material itself (virgin polymer pellets), while another part is adjusted during processing to meet the functional requirements of the final product. Common additives include inorganic fillers (e.g., glass fibers, calcium carbonate, talc, carbon black), stabilizers, antioxidants, flame retardants, and color pigments. The same polymer may be compounded with different additives and filler concentrations, depending on the needs of the converter [6].

Another critical aspect is that plastic waste is aged material, and the polymer may be partially degraded due to wear and environmental exposure. Typically, this leads to oxidation phenomena, resulting in lower molecular weights and a decrease or loss of mechanical properties.

Process-related issues mostly stem from the inevitable partial thermal degradation during processing, which makes it necessary to add additional stabilizers (typically antioxidants) during the recycling process. The inherent limitations of mechanical/thermal recycling affect the properties of the recycled polymer in the following ways:

- The recycled material is different from virgin polymer: while it may be similar in chemical characteristics and performance, it is effectively a new material.
- Recycled polymers can vary from batch to batch, due to inconsistent and hard-to-control input materials. In some cases, inhomogeneities may be present even from pellet to pellet, or within a single pellet (especially if foreign inclusions are present, such as metal flakes).

To ensure proper use of secondary raw materials obtained from plastic waste recycling, it is essential to have a comprehensive understanding of their physicochemical properties [3]. This enables comparisons with the reference virgin polymer and with other recycled polymers available on the market.

Infrared (IR) spectroscopy has been employed since the earliest polymer syntheses as a fundamental tool for characterizing these materials [7,8,9]. Thanks to its ability to recognize molecular structures—the infrared region between 1800 and 400 cm^−1^ is in fact known as the fingerprint region—IR spectroscopy is widely used in chemical synthesis laboratories, both for the identification of pure compounds and mixtures, and in structural characterization of molecular materials [10,11,12,13,14]. IR spectroscopy also enables the detection of compounds arising from degradation phenomena and contaminant species.

Nevertheless, the potential of IR spectroscopy extends far beyond chemical identification—nowadays often automated through spectral databases or correlative analyses. Spectral studies have significantly advanced our understanding of molecular physics, beginning with early investigations of vibrational dynamics and the development of vibrational transition theory [15,16,17]. More recently, major progress in the interpretation of IR spectra has been achieved through first-principles modeling of molecular structures and their spectroscopic response (e.g., DFT) [18,19]. Building on these foundations, polymer IR spectroscopy has evolved by combining well-established empirical correlations with theoretical studies of molecular vibrations extended to the case of ideally infinite linear polymers and polymer crystals [20,21,22,23]. A substantial body of theoretical and experimental work is now available [8,9,22,23,24,25,26,27,28], enabling critical interpretation of polymer spectroscopic responses and providing valuable tools for the analysis of complex materials, including recycled polymers.

Below is a summary of the chemical and structural information on polymer materials that can be obtained through IR spectroscopy, several aspects of which will be discussed in the following sections:

- Chemical identification, including additives: especially useful for studying unknown formulations, can achieve quantitative diagnostics through calibration curves based on reference samples of known composition.
- Diagnosis of chemical modifications: important for monitoring degradation during production (e.g., extrusion, molding, spinning) or aging under various environmental conditions.
- Structural characterization: identifying molecular conformation, degree of crystallinity, and structural defects in semi-crystalline polymers.
- Morphological analysis: distinguishing amorphous from crystalline phases, identifying crystalline polymorphs, quantifying their relative fractions, and recognizing polymer chain orientation, as in fibrous materials.
- Thermal transformation analysis: monitoring phase transitions (e.g., melting) and post-treatment effects (annealing, thermal cross-linking) by recording spectra during controlled heating/cooling cycles, thereby reconstructing the material’s thermal history.

Most of this information is obtained by analyzing the frequencies and intensities of IR absorption bands associated with vibrational transitions. In addition, band-shape analysis often provides insights into material morphology (e.g., amorphous vs. crystalline domains) and its evolution (e.g., during ageing).

However, not all structural information required for a detailed characterization of recycled polymers can be obtained through IR spectroscopy. A particularly relevant limitation is its low sensitivity to molecular weight distributions. During mechanical recycling of polymers, the average molecular weight often decreases significantly due to thermal degradation, leading to the formation of low-molecular-weight components. While IR can reveal such effects indirectly—through morphological changes or by detecting terminal functional groups in the presence of substantial low-molecular-weight fractions—detailed information on molecular weight polydispersity remains difficult to access. By contrast, oxidative processes that produce oxygen-containing functional groups can be more readily detected through their characteristic absorptions.

Another limitation concerns the measurement setup, which often requires specific sample preparation tailored to spectral acquisition—a non-negligible drawback when rapid diagnostics are needed. In this respect, Raman spectroscopy should be noted as a complementary technique to IR for probing vibrational transitions (and thus molecular structure) in polymers. Although generally less sensitive to chemical and structural defects, Raman spectroscopy typically poses fewer sampling challenges and, in some cases, may represent a valid alternative to IR spectroscopy [29].

In this paper, we discuss the potential of IR spectroscopy in the context of polymer recycling, explicitly addressing its contributions in light of the various complexities that may arise. Particular attention is given to methodological aspects, and through selected examples, we propose guidelines for the critical interpretation and use of spectroscopic markers that are not typically considered in this field.

This study outlines the steps required to extend IR spectroscopy beyond its current role in the sorting of recyclable polymers. The focus is on identifying key challenges in the development and use of recycled materials, assessing the ability of IR spectroscopy to address them, and refining methodologies to extract reliable structural and chemical information.

The discussion includes examples from the spectroscopic analysis of commercially sourced recycled polymer pellets, as well as reference spectra from virgin polymers. A case study on the quantitative determination of polypropylene content—which is frequently present in recycled polyethylene samples—will also be presented.

## 2. Materials and Methods

### 2.1. Materials

Several commercial polymer samples were analyzed, including high-density polyethylene (HDPE), low-density polyethylene (LDPE), isotactic polypropylene (PP), polyamide (PA), polystyrene (PS), polyvinyl chloride (PVC), polyethylene terephthalate (PET), and recycled HDPE pellets (R-HDPE) from different manufacturers.

Isotactic PP and HDPE purchased from Aldrich were used as reference materials for constructing the PP/HDPE calibration curve to investigate the PP concentration in R-HDPE samples. Pure HDPE and isotactic PP pellets were mechanically ground using a steel-blade blender under liquid nitrogen to prevent thermal degradation. The resulting powder was sieved through a ~100 µm mesh to isolate the finest fraction. Six mixtures with varying HDPE/PP compositions were then prepared by weighing the appropriate amounts of each component. Approximately 150 mg of each mixture was pressed into pellets at 10 tons/cm^2^ for 2 min. Thin films (~100 µm thick) were subsequently obtained by compression molding at 200 °C. Three replicate films were prepared for each composition to ensure reproducibility.

Films of R-HDPE samples were produced following the same procedure, by compression molding the recycled polymer pellets at 200 °C.

For the outdoor weathering study, two polymers were considered: (i) a carbon monoxide–ethylene copolymer commercialized under the trade name Carilon D 26 HM100 (Shell Italia S.p.A., Milan, Italy), and (ii) a commercial isotactic polypropylene (Moplen^®^ T 30 S, Monteshell, Marostica, Italy). Both polymers were used as received. Carilon^®^ specimens were prepared as thin films (40–80 μm thick) by compression molding pellets at 225 °C, whereas Moplen^®^ specimens were prepared as 2 mm thick plates by compression molding pellets at 190 °C. Films and plates were mounted without stress on wooden frames tilted at 45° relative to the horizon and exposed outdoors, facing southwest, on a terrace approximately 20 m above ground in Messina, Italy.

### 2.2. Spectroscopic Characterization

Transmission FTIR. Transmission-mode infrared (IR) spectra of recycled polymers, PP/HDPE samples, and R-HDPE thin films were recorded on a Nicolet 6700 Fourier-transform infrared (FTIR) (Thermo Fisher Scientific, Waltham, MA, USA) spectrometer in the range 400–6500 cm^−1^ (128 scans, 4 cm^−1^ resolution).

ATR-FTIR. Attenuated Total Reflectance (ATR) spectra of polymers and outdoor-exposed specimens of Carilon^®^ and Moplen^®^ were collected using a Thermo Electron Continuµm IR microscope coupled to the same Nicolet 6700 FTIR spectrometer, equipped with a slide-on single-bounce ATR accessory with a silicon crystal (128 scans, 4 cm^−1^ resolution). ATR spectra of outdoor-exposed samples and polymers were intensity-corrected using the ATR correction function implemented in the OMNIC software (version 8.2.0.387, Thermo Fisher Scientific Inc., Waltham, MA, USA) to facilitate comparison with transmission spectra. No additional post-processing (e.g., baseline correction, spectra smoothing) was applied to the spectra reported in this study.

## 3. Results and Discussion

### 3.1. General Characteristics of Polymeric Materials and Their IR Spectra

The discussion of polymer spectra must take into account the peculiar characteristics of these materials [30,31] and spectroscopic features [8,9,24,25,26,27,28] that are independent of whether the material is a virgin polymer or a second-raw material obtained through recycling processes.

- A polymeric material is often a formulation: in addition to the polymer itself, it contains additives that meet processing requirements and ensure the durability of the final product. Additives may provide chemical stability, while fillers can modify mechanical properties (e.g., reinforcing glass fibers) or electrical properties (e.g., graphene particles). Many polymeric materials intended for specific technological applications consist of blends of two or more polymers or copolymers with variable composition. The resulting IR spectrum is therefore a superposition of the spectral responses of all components and cannot perfectly match that of a pure homopolymer or even samples of the same polymer/copolymer that differ in additive type and/or concentration.
- Automated identification (typically using spectral libraries) is an effective and valuable method for determining the polymer family, especially when combined with supervised analysis. This is commonly employed for plastic sorting in recycling processes. However, especially for semi-crystalline polymers, sample preparation can influence morphology [30,31], resulting in spectral differences compared to literature references or database entries. Furthermore, the measurement technique—such as transmission, attenuated total reflectance (ATR), or reflection—can significantly alter the spectral pattern (band shape and intensity), even when spectra are mathematically converted to a common absorbance (or transmittance) scale. Note that absorbance conversion—except in transmission mode—is based on theoretical models and assumptions that are never perfectly met in practice.
- Effective use of spectral libraries often requires data post-processing: baseline correction and subtraction of solvent, substrate, or contamination bands may be necessary. Spectral comparison also requires proper normalization procedures, typically based on normalizing absorbance intensity to a reference band. These processes are not automatic and require expertise, as they may introduce artifacts.

### 3.2. Techniques and Measurement Setups

Polymeric materials are generally solid samples, and when analyzing their structure and morphology, it is essential to use an appropriate experimental setup and/or properly prepare the sample. Several measurement techniques are well documented in the specialized literature [13,14,27]. In this section, we briefly list the most commonly used methods, highlighting which types of polymeric samples they are best suited for. In addition to transmission experiments, which directly yield the absorption spectrum, alternative setups exploit different physical phenomena—primarily reflection and scattering—associated with IR photons absorption. These methods can reconstruct an absorbance spectrum through appropriate mathematical processing. However, as such procedures often introduce some distortion in the spectral profile, it is considered best practice—particularly for quantitative analysis—to compare spectra obtained using the same technique and experimental setup. For solid polymer samples, the following methods are available:

Transmission measurements. Suitable samples include fine polymer powders dispersed in KBr pellets; thin films deposited on a transparent substrate (e.g., KBr or ZnSe windows); thin films deposited on a reflective substrate (e.g., aluminum, gold, silicon wafer). In the last case, the setup is called double transmission, as the IR beam passes through the sample twice (incident and reflected beam) before reaching the detector. By compression in a diamond anvil cell (DAC) accessory, very small polymer particles can be prepared as a thin film suitable for transmission-mode analysis. It should be noted, however, that the application of high pressure may induce morphological changes in the material.

Reflection measurements comprise

- ATR: One of the most convenient techniques for polymeric materials. It detects absorption of the evanescent wave generated at the surface of a high-refractive-index crystal in contact with the sample. Since surface layers often contain additives (e.g., slip agents, release agents), for bulk analysis, it may be necessary to remove the surface layer or section the sample. With special ATR accessories, direct measurements on finished products are also possible.
- Specular reflection: Suitable for polymeric materials with good reflectivity (also depending on surface finish). The sample must be thick enough to prevent the collection of transmitted photons (as in double transmission setups).
- Diffuse Reflectance (DRIFT): Captures diffusely scattered IR radiation over a large solid angle, from which the IR spectrum is obtained. Applicable to samples with high IR scattering (e.g., powders or granular solids), where transmitted IR is negligible and specular reflection is weak.

### 3.3. Spectral Analysis

There are well-established procedures used by companies and manufacturers for quality control of various polymeric materials via IR analysis. Due to their applied nature and confidentiality, these methods and algorithms are rarely detailed in the scientific literature, and general accessible guidelines are lacking. This section aims to provide practical methodological guidance for addressing the challenges in studying recycled or recyclable polymers, supported by selected examples. IR spectroscopy can assist in (i) separation of polymeric materials for recycling and (ii) analysis/evaluation of recycled material (second-raw material).

#### 3.3.1. Sorting

IR analysis, particularly in the near-infrared (NIR) region, is a well-established method for rapid sorting of polymers to be recycled. These applications often focus on identifying the specific polymer class (e.g., polyethylene, polypropylene, polyamide, polyethylene terephthalate, polyvynil chloride, etc.). Identification is enabled by the spectroscopic “fingerprints”—intense absorption bands corresponding to the fundamental vibrational modes of the polymer. In this context, in addition to supervised sorting by operators, automated techniques such as NIR/MIR hyperspectral imaging can be employed on industrial lines [6]. Effective sorting should not only selectively identify a specific polymer but also minimize contamination with other polymeric species that may or may not be miscible with the primary polymer, as such contamination can degrade material properties [32,33,34,35].

The sorting challenge is particularly relevant for post-consumer recycled material, which additionally requires preliminary removal of non-polymeric components (metal fragments, paper, etc.). In the case of post-industrial waste, the polymer and its formulation should, in principle, already be known—especially when the scraps are returned directly to the recycling company.

**Figure 1:**
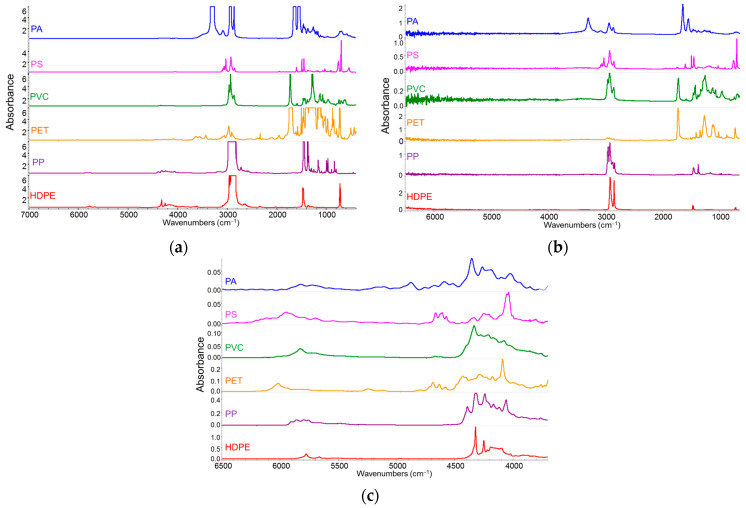
IR spectra of samples (thick films) of commercial polymeric materials (polyamide, PA—blue line; polystyrene, PS—magenta lin; polyvinyl chloride, PVC—green line; polyethylene terephthalate—yellow line, PET; polypropylene, PP—purple line; high-density polyethylene, HDPE—red line). (**a**) Overview of IR-NIR spectra recorded in transmission mode, showing saturation of intense absorption bands (in the 3500–800 cm^−1^ region) corresponding to fundamental transitions. (**b**) Overview of IR-NIR spectra recorded in ATR mode. ATR spectra allow observation of absorption bands corresponding to fundamental transitions without saturation effects; (**c**) transmission spectra, zoomed into the NIR region (3500–6500 cm^−1^), where overtone and combination bands are observed. Due to the weak intensity of these bands, even in thick samples, the absorbance values are suitable for quantitative diagnostics.

Figure 1 shows a comparison of NIR-IR spectra of common polymers, some of which are currently subject to recycling. The spectra clearly demonstrate that identification is possible by detecting intense and selective absorption peaks specific to each polymer. Spectra were recorded on manufactured samples (thick films) using both transmission mode (Figure 1a) and ATR mode (Figure 1b). The intense absorption bands in the mid-IR region—corresponding to fundamental transitions (3500–400 cm^−1^)—enable highly effective polymer identification, which can be performed either automatically using spectral libraries or through empirical correlations based on characteristic group frequencies [10].

It is important to note that spectra acquired in transmission mode (Figure 1a) show saturation effects in the intense fundamental bands, which hinder reliable identification. In contrast, spectra recorded in ATR mode overcome this limitation and can be used to identify the main polymeric component in the material by focusing on the most intense bands. Unlike ATR spectra, where NIR transitions are extremely weak and mostly indistinguishable from experimental noise, transmission spectra of films exhibit a good signal in the NIR region. In this case, overtone and combination bands can be used for both material sorting and quantitative diagnostics (see Section 3.3.3).

**Figure 2:**
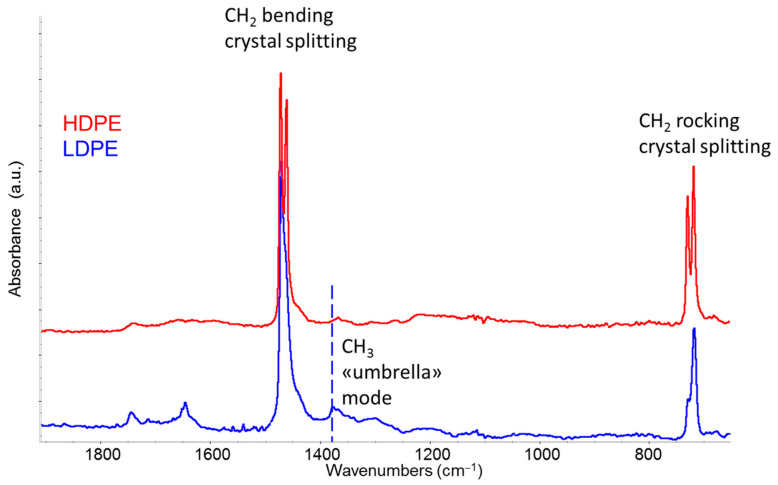
Comparison of IR-ATR spectra of HDPE (red line) and LDPE (blue line) in the fingerprint spectral region. Rocking and bending bands of CH_2_ groups, which exhibit more pronounced crystal-splitting in the case of HDPE, are highlighted. The dashed line indicates the bending “umbrella” band (1375 cm^−1^) associated with the presence of methyl groups, which is particularly prominent in LDPE.

Polyethylene is one of the most abundant polymers found in post-consumer plastic waste. It is widely used in packaging—primarily as low-density polyethylene (LDPE)—and, in the form of high-density polyethylene (HDPE), in a variety of manufactured products (containers, furniture components, toys, pipes, sheets) where enhanced mechanical properties are required. During the recycling process, it is important to separate HDPE from LDPE, and IR spectroscopy is a powerful tool for this purpose, provided that one focuses on specific spectral features that support accurate classification. Figure 2 shows a comparison between the ATR spectra of two samples of virgin HDPE and LDPE.

The differences between HDPE and LDPE relate to the relative abundance of amorphous and crystalline phases. These differences are reflected in a more pronounced crystal splitting in the rocking and bending bands of HDPE, as well as in the intensification of absorption signals associated with polymer chains in distorted conformations in the CH_2_ wagging region 1300–1370 cm^−1^ in the case of LDPE [25,28]. An additional marker of LDPE is the appearance of a very characteristic band at 1375 cm^−1^, attributed to the symmetric bending of the methyl group—often referred as “umbrella mode” [25,28]. This band gains significant intensity in the presence of the branching of the polymer’s main chain, which ends with CH_3_ groups. These branches are typical of LDPE but are less frequent in HDPE due to the tighter control of the molecular structure in catalyzed synthesis.

Any analysis based solely on the observation of the fundamental transitions of the main polymer cannot exclude the presence of contaminants, additives, or other polymers in small percentages. We will explore these aspects in greater detail in Section 3.3.2 and Section 3.3.3, by analyzing recycled polymer pellets, where mixtures of similar polymers—such as PE and PP, or HDPE and LDPE—represent one of the most common issues.

In the context of analyzing material intended for recycling, IR analysis based on the identification of oxidation markers—typically the C=O stretching band around 1700 cm^−1^—can be useful [36,37,38]. More importantly, the analysis of band shapes can provide insights into the aging and degradation levels of the materials. This information could be used as a criterion to discard low-quality materials before undergoing mechanical recycling.

#### 3.3.2. Comparative Analysis of Recycled Polymers: R-HDPE

The IR analysis of recycled polymeric materials—usually supplied in pellet form to manufacturers—requires appropriate experimental strategies, and several examples of application of IR spectroscopy to specific cases are reported in the literature [37,38,39,40].

Depending on the type of information needed, it may be preferable to perform ATR measurements or transmission measurements on films obtained by melting and compressing the pellets. Quantitative determinations require the development of calibration methods using model formulations prepared ad hoc, as will be illustrated in a case study (Section 3.3.3). In this section, we present some comparisons between IR spectra of recycled HDPE pellets (R-HDPE), showing how a supervised analysis of ATR spectra can already highlight significant differences in chemical composition and structure.

**Figure 3:**
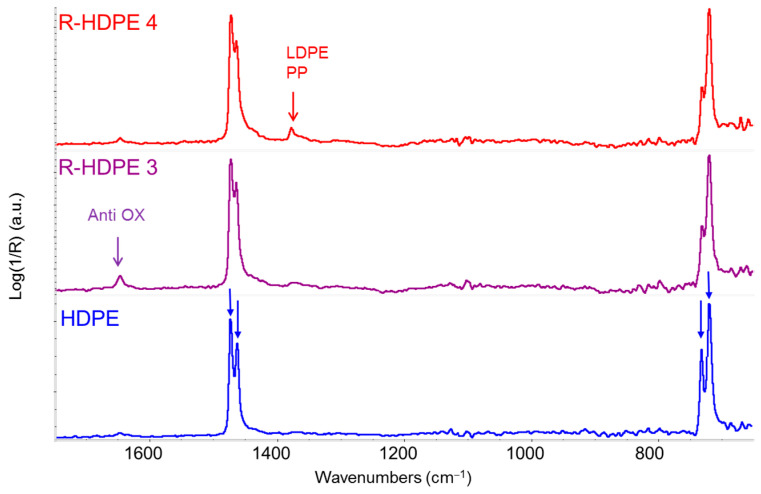
Comparison of ATR infrared spectra (1700–650 cm^−1^ region) of two commercial recycled HDPE (R-HDPE) pellets from different sources (purple and red lines) and a virgin HDPE sample (blue line). The crystal splitting of the CH_2_ bending bands (1463–1473 cm^−1^) and the CH_2_ rocking band (722–730 cm^−1^) is highlighted by blue arrows in the spectrum of virgin HDPE, which displays well-resolved doublets compared to the spectra of R-HDPE samples.

Figure 3 shows the IR (ATR) spectra of two R-HDPE samples obtained from different manufacturers. Comparison with the spectrum of a virgin HDPE pellet reveals that each R-HDPE sample is distinct, and all differ noticeably from the virgin material. The zoomed-in view of the fingerprint region highlights the bending and rocking bands, which exhibit characteristic crystalline splitting (doublets at 1463–1473 cm^−1^ and 722–730 cm^−1^, respectively)—a hallmark of the crystalline phase [25,28]. These doublets are clearly visible in the IR spectrum of virgin HDPE, where the degree of crystallinity is high (typically around 60%). In contrast, they are less well resolved in the spectra of recycled pellets, due to the contribution of the amorphous phase. The latter gives rise to a broader band component between the two peaks of each doublet, thus indicating the reduced crystallinity of R-HDPE.

**Figure 4:**
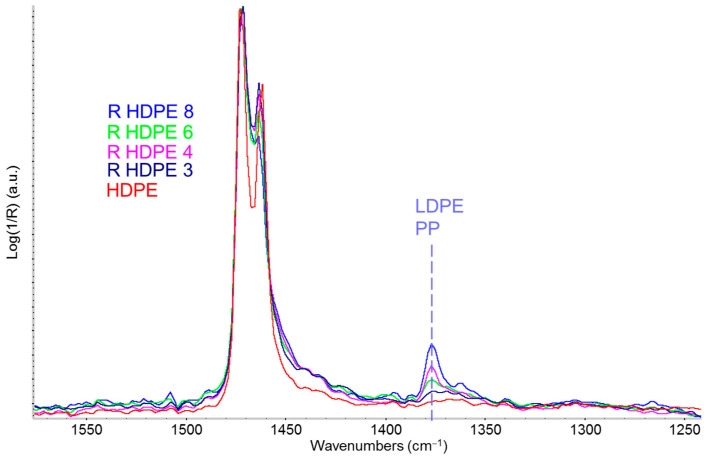
Comparison of ATR infrared spectra of several commercial recycled HDPE (R-HDPE) pellets from different sources and a virgin HDPE sample (red line) in the 1600–1250 cm^−1^ region.

In Figure 4, a zoomed-in view of the IR spectra highlights the marker band of the methyl group at 1375 cm^−1^ (CH_3_ umbrella mode), which appears particularly intense in sample 4 (red line). This could be attributed to a significant presence of LDPE within the R-HDPE sample. However, since PP also exhibits a strong CH_3_ bending band at nearly the same wavenumber, it is also plausible that sample 4 contains a certain amount of PP. Notably, the varying intensity of the band at 1650 cm^−1^ (Figure 3) attributed to a common antioxidant, indicates different additive concentrations across the three samples.

The first conclusion drawn from the analysis of the R-HDPE samples in Figure 4 is that the label R-HDPE does not guarantee a crystallinity level comparable to that of virgin HDPE. This is likely because the polymeric fraction in R-HDPE consists of a blend of HDPE and some amount of LDPE and/or it contains a fraction of PP. It is well known that the degree of crystallinity strongly influences the mechanical performance of polymer materials [30,31].

**Figure 5:**
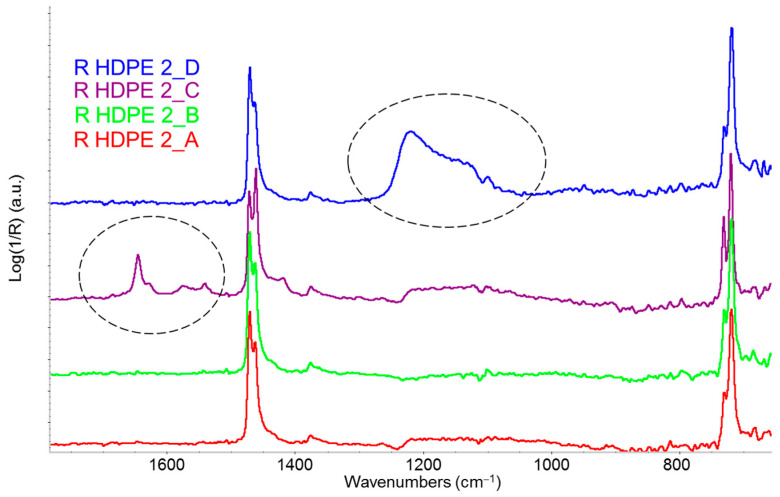
IR-ATR spectra (fingerprint region) of various pellets from the same R-HDPE batch. Absorptions related to additives or contamination are highlighted by dashed circles.

Another important observation arises from comparing ATR spectra of different granules within the same R-HDPE batch (Figure 5). Some granules, when examined under an optical microscope, reveal fragments of extraneous materials. IR analysis confirms that different pellets exhibit varying chemical compositions. Spectral subtraction and comparison with reference spectra from a database reveal that sample A contains common anti-tack additives on the pellet surface.

#### 3.3.3. Detection and Quantification of Polypropylene in R-HDPE Samples

**Figure 6:**
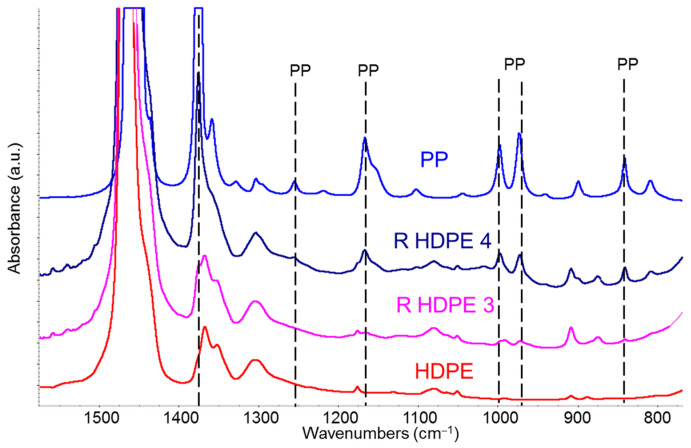
Comparison of IR spectra of R-HDPE samples with those of virgin HDPE (red line) and virgin polypropylene (PP, blue line). Spectra were recorded in transmission mode on films obtained by compression moulding from the pellets. The dashed vertical lines highlight characteristic absorption peaks of PP. Spectra are vertically staked for sake of clarity.

Figure 6 shows a comparison that highlights the presence of polypropylene within R-HDPE samples. The spectra of the recycled polymer, recorded in transmission mode on a film obtained by compression molding from the pellets, exhibit the typical saturation of polyethylene’s fundamental absorption bands. The spectral range shown in Figure 6 clearly illustrates this saturation phenomenon for the fundamental CH_2_ bending doublet at 1463–1473 cm^−1^. However, it also clearly reveals several weak absorption features (dashed vertical lines in Figure 6) attributable to the presence of a polypropylene fraction, likely introduced as contamination during the separation stage of the recycling process.

The presence of isotactic polypropylene (PP) in R-HDPE samples is quite common due to the intrinsic challenges during the sorting process. While small amounts of PP may have beneficial effects on the mechanical and rheological properties of the material, it is crucial to reliably control the PP content to ensure consistent material properties when using different commercial R-HDPE products or different batches from the same manufacturer. To address this, we developed methods based on intensity ratios of selected marker bands for HDPE and PP, enabling quantification of the PP weight percentage in the range of 0–15%. Interestingly, there is no single optimal strategy for selecting marker bands for this analysis. For example, absorption features from certain additives can mask useful PP and HDPE marker bands in the mid-IR region. In such cases, the near-infrared (NIR) region provides more informative spectral data. Reference calibration curves were obtained using lab-prepared samples consisting of known weight ratios of virgin PP and HDPE.

In order to use IR spectroscopy for the quantitative determination of polypropylene (PP) content in commercial recycled HDPE (R-HDPE), transmission IR measurements were performed on thin films of HDPE/PP blends prepared according to the method described in Section 2. Three films were prepared and analyzed for each sample. Table 1 reports the composition of the different reference samples, labeled with alphabetical letters (samples a–f).

**Figure 7:**
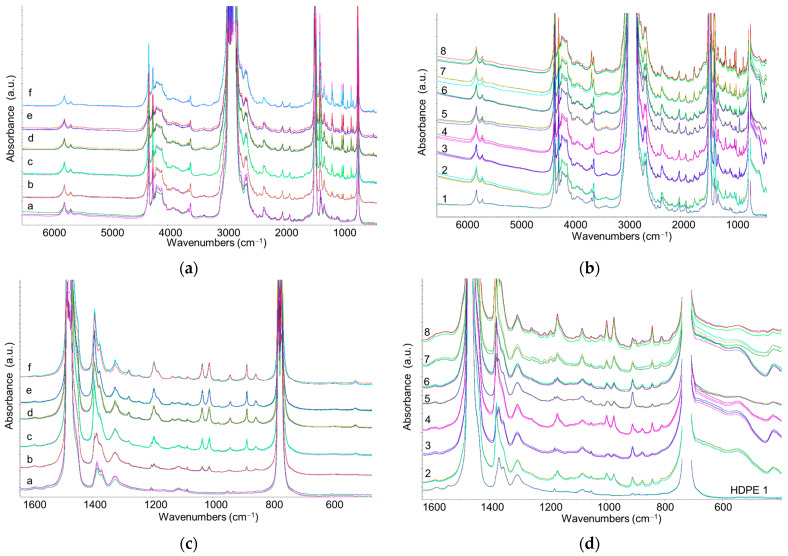
IR–NIR transmission spectra of (**a**) reference samples (HDPE/PP mixtures, samples a–f) and (**b**) R-HDPE samples (samples 2–8) together with commercial HDPE (sample 1). Enlarged views of the IR spectra in the fingerprint region: (**c**) reference samples and (**d**) R-HDPE samples.

Several commercial recycled PE samples (designated as samples 2–8), together with a commercial virgin HDPE (sample 1), were processed into thin films directly from the pellets. IR spectra of the reference samples (a–f) and R-HDPE samples were recorded in the 350–7000 cm^−1^ range to capture absorptions corresponding to first overtones and combination bands of fundamental transitions in the NIR region (Figure 7). As usual, the strong fundamental absorption bands of polyethylene exhibit saturation effects in the spectra recorded in transmission mode; however, PP features are clearly detectable, even in reference samples with low PP content (Figure 7c). In particular, PP features are also observed as weak but distinct peaks in the spectra of R-HDPE samples (Figure 7d).

**Figure 8:**
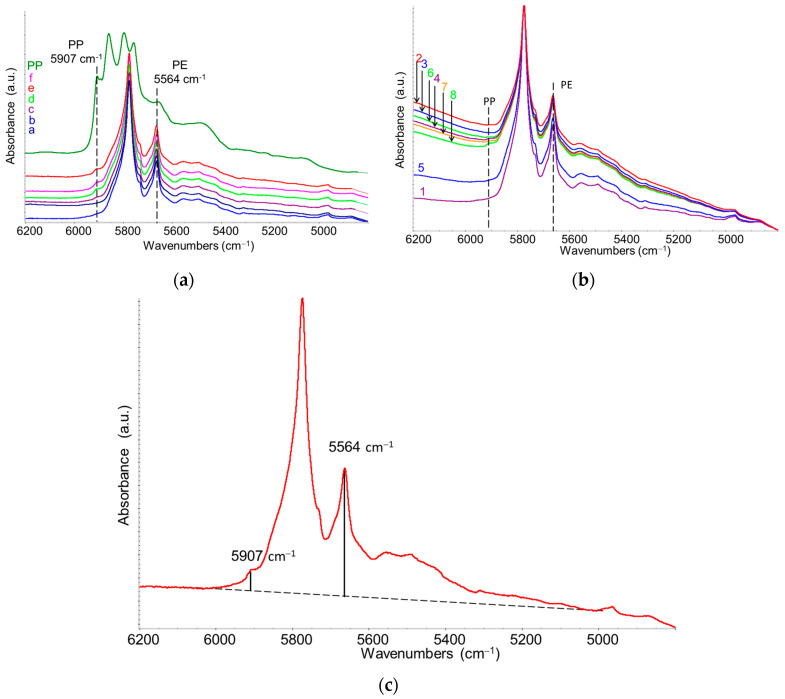
NIR spectra (6200–4800 cm^−1^ region, transmission mode) of (**a**) reference samples (HDPE–PP mixtures, samples a–f) together with the reference spectrum of isotactic PP (top, green line) and (**b**) R-HDPE, samples 2–8 and commercial HDPE, sample 1. Panel (**c**) shows the procedure used to measure the height of representative peaks of HDPE and PP, which are highlighted by vertical dashed lines in panel (**a**,**b**). The spectra in panels (**a**,**b**) have been vertically stacked for clarity.

For R-HDPE samples, the simultaneous presence of absorption bands from polymers, fillers, and additives makes the spectral region very crowded and masks several weak HDPE bands (see Figure 7c,d) that could otherwise be used to quantify the relative PP/HDPE content. Therefore, we focused our analysis on the NIR region (4000–7000 cm^−1^), where overtone and combination bands are observed (Figure 8). The NIR analysis offers several additional advantages:

1. Absorption intensities of overtone and combination bands are much weaker than fundamental transitions, reducing bands saturation effects even in relatively thick films. In fact, thicker samples can enhance the visibility of these weak bands.
2. Additives and fillers exhibit very weak absorptions in the NIR, making their contribution negligible compared to the main polymer component. Hence, the obtained information primarily reflects the bulk polymer.
3. Transmission mode ensures analysis of the bulk material, preventing overestimation of surface chemical species possibly present on pellets.

For the construction of the calibration curve, the HDPE marker band at 5664 cm^−1^ (1797 nm) was selected, corresponding to the first overtone of symmetric C–H stretching vibration of the methylene (CH_2_) group [41]. As for PP, the marker band at 5907 cm^−1^ (1694 nm) was chosen, corresponding to the first overtone of asymmetric C–H stretching vibration of the methyl (CH_3_) group. These two bands were selected due to their rather good separation and easy identification in both reference blend samples and commercial recycled HDPE samples (Figure 8a,b).

**Figure 9:**
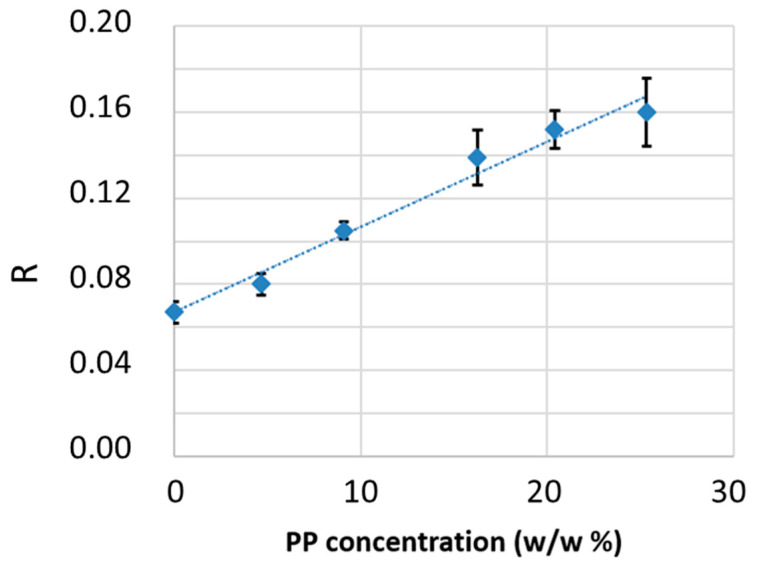
Calibration curve based on the ratio $R=\frac{I_{5907}}{I_{5564}}$ measured from the reference samples.

The peak height of the bands at 5664 cm^−1^ and 5907 cm^−1^ was measured after baseline correction by drawing a straight line between 6000 and 5000 cm^−1^ (Figure 8c). For each concentration, three spectra from three different films were recorded, and the average peak intensity ratio of the marker bands was calculated and associated with the relative concentration. Numerical values are reported in Table 1, and the trend is shown in the plot in Figure 9. As predicted by the Lambert–Beer law, under the assumption of no interactions between the HDPE and PP phases, a linear correlation between the intensity ratio and the PP relative concentration was observed. From these data, a linear regression allowed deriving a calibration function describing PP relative concentration $C_{PP/PE}$ (*w*/*w*%) as a function of the band intensity ratio *R* = *I*_5907_/*I*_5564_.

(1) $$C_{PP/PE}=\left(\frac{I_{5907}}{I_{5564}}-0.067\right)\times \frac{1}{0.004}$$

This calibration curve was used to determine the PP content in commercial recycled HDPE samples. Spectra were recorded on three films per R-HDPE sample (spectra are reported in Figure 8). The average values of *R* = *I*_5907_/*I*_5564_ and the estimated PP weight percentages from the calibration curve are summarized in Table 2.

In conclusion, the careful preparation of reference samples with known PP concentrations in HDPE enabled the establishment of a reliable calibration curve useful for detecting small percentages of PP. The method can discriminate samples with vanishing PP levels: sample 1, a commercial sample marketed as virgin HDPE, showed a negligible estimated PP content (~0.3%), confirmed by supervised spectral analysis in the fingerprint region (950–1000 cm^−1^), where no PP-related absorptions were observed. All other samples (R-HDPE) showed clear PP signals in the fingerprint region, thus containing estimated PP relative percentages higher than 1.5%. No standardized reference values are available for the allowable PP content in R-HDPE. While an ideal sample would contain no PP, this is unattainable due to the inherent limitations of sorting processes. In practice, the acceptable PP threshold should be defined according to the performance requirements of the final application. IR spectroscopy offers a reliable tool for quality control at the procurement stage, which is particularly relevant given the significant variability observed across batches.

#### 3.3.4. IR Detection of Polymer Degradation

As a final example, we illustrate how IR analysis can assist in diagnosing plastic degradation. Two previous studies [36,42] on the outdoor aging of commercial, non-recycled polymeric materials are presented, with the aim of demonstrating the ability of IR spectroscopy to monitor polymer aging processes in terms of structural and morphological modifications. To the best of our knowledge, no shared guidelines currently exist for the investigation of aging in recycled polymeric materials. Spectroscopic analysis of these materials, when compared with that of virgin reference samples, can provide valuable insights into the distinct—presumably more accelerated—evolution of the molecular and supramolecular structures of recycled polymers, even at a stage when deterioration of mechanical properties is not yet detectable. The examples reported here indicate that IR spectroscopy should be considered alongside mechanical performance testing when assessing the durability of recycled materials.

**Figure 10:**
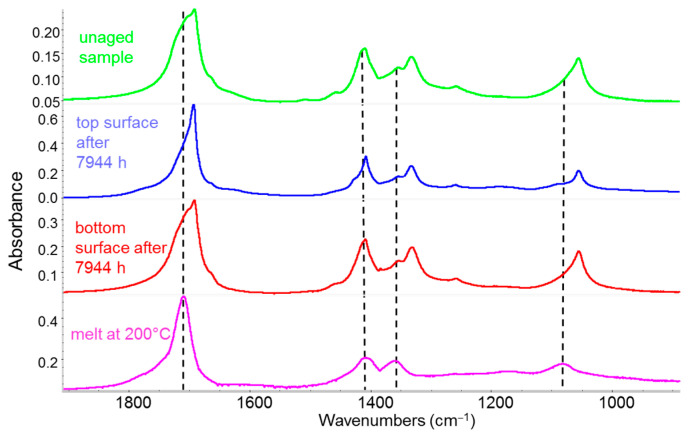
IR spectra of a Carilon^®^ plate. Green line: spectrum of the unaged sample; blue line: spectrum of the top surface, directly exposed to sunlight, after 7944 h of outdoor weathering; red line: spectrum of the bottom surface, not directly exposed to sunlight, after 7944 h of outdoor weathering; magenta line: spectrum of the melted sample, showing spectral features associated with the amorphous phase. The dashed lines mark the peak positions of the broad absorption band components associated with the amorphous phase.

The first case study [42] focuses on the effects of outdoor weathering on a specialized polymer—an alternating ethylene–carbon monoxide copolymer, commercialized under the trade name Carilon^®^, a semi-crystalline polymer employed in the automotive industry. Figure 10 shows a comparison between a pristine, unaged Carilon^®^ sample and one exposed outdoors for 331 consecutive days. For the latter, IR spectra (collected in ATR mode) are presented for both surfaces of the exposed specimen. For comparison, the spectrum of the same polymer in the molten state is also reported (Figure 10, bottom). This spectrum reproduces the absorption band shapes of the amorphous phase, with the peak position highlighted by the dashed lines. Infrared spectra reveal that all IR bands of the pristine polymer, which represent a convolution of absorptions from both amorphous and crystalline domains, become sharper with increased exposure time. This observation indicates that sunlight promotes the loss of the amorphous phase, typically characterized by broad absorption features.

What remains—especially when analyzing the upper surface of the sample, which was directly exposed to sunlight—are small polymer crystals no longer embedded in the amorphous matrix [42]. This structural change results in a material that is significantly more brittle [42]. Furthermore, a detailed examination of the IR spectra reveals the appearance of new bands showing a progressive intensity increase over time, enabling the identification of specific degradation products [42].

**Figure 11:**
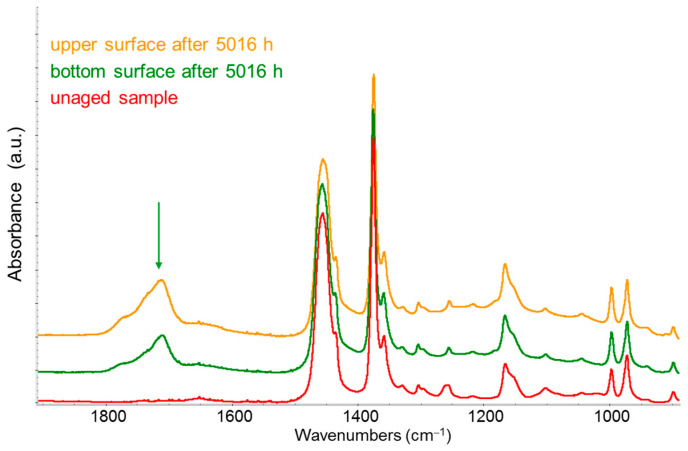
IR spectra of an isotactic polypropylene plate. Red line: spectrum of the unaged sample; green line: spectrum of the bottom surface, not directly exposed to sunlight, after 5016 h of outdoor weathering; yellow line: spectrum of the top surface, directly exposed to sunlight, after 5016 h of outdoor weathering. The samples exposed outdoors show the characteristic C=O stretching band around 1700 cm^−1^ (green arrow), whose intensity indicates greater polymer oxidation on the surface directly exposed to sunlight.

Another example of polymer degradation detection using IR spectroscopy is illustrated in Figure 11, which shows the IR spectra of commercial isotactic polypropylene (PP) plates exposed to outdoor conditions for a prolonged period (up to approximately 200 days) [36]. The main evidence in this case is the appearance of a new broad band around 1700 cm^−1^—corresponding to the C=O stretching mode—arising from polymer oxidation.

While the two examples discussed above refer to virgin polymers, the same type of analysis could be applied to recycled materials to assess their stability over time through accelerated ageing tests in a controlled environment.

## 4. Conclusions

We have presented numerous comparisons and analyses of infrared spectra of recycled polyethylene, one of the most abundant polymeric materials found in plastic waste. Detailed analysis of these spectra, together with a comparison to infrared spectra of reference samples, has demonstrated that, by carefully optimizing the experimental setup and focusing on the most suitable spectral region, it is possible to obtain accurate information about the chemical composition and molecular structure of the material. Expert use of IR spectroscopy, which can be partially automated, provides valuable solutions both in the sorting and classification phase of recyclable materials and for assessing the quality and reproducibility of the material obtained from recycling processes. Below, we summarize some key insights that have emerged from this study:

- The use of IR for materials characterization provides a detailed description of chemical composition, molecular structure, and morphology. Different materials or issues may require choosing different experimental setups. Standardization of protocols remains a challenge.
- R-materials are different from virgin ones. Available IR techniques allow the identification of several differences between R-polymer materials and virgin polymers.
- Characterization of R-polymers can suggest strategies to bridge the gap and/or develop new structure–property correlations with application perspectives in mind.
- The durability of R-polymers should be evaluated through appropriate accelerated weathering or ageing tests, followed by structural analysis, e.g., using IR.

These conclusions are supported by several examples, mostly concerning the case of polyethylene and polyethylene/polypropylene blends, which represent a significant fraction of plastic waste subjected to recycling. These polymers are semi-crystalline, and structural investigation by means of IR spectroscopy is particularly relevant, as it allows one to probe the phenomena related to the interplay between the amorphous and crystalline phases, which have a strong influence on the mechanical properties of the polymer materials.

The methodologies illustrated here can be extended to other classes of recyclable polymers, and in particular to all semi-crystalline polymers. In the case of fully amorphous polymers, IR spectroscopy can be especially useful for chemical diagnostics, particularly for the identification of additives and contaminants present in recycled materials, as well as for the detection of oxidative processes.

## Figures and Tables

**Table 1:** Composition of reference samples prepared by mixing virgin HDPE and PP powders and NIR data (intensity ratios) used to build the calibration curve for estimating the PP fraction in R-HDPE samples.

Sample	PP [mg]	HDPE [mg]	$C_{PP/PE}$ $\left(\frac{w}{w}\%\right)$	$R\;=\;\frac{I_{5907}}{I_{5564}}$
a	0.0	160.5	0.00	0.067
b	7.0	149.4	4.69	0.080
c	13.8	151.5	9.11	0.105
d	19.6	120.5	16.27	0.139
e	27.8	136.2	20.41	0.152
f	26.9	106.2	25.33	0.160

**Table 2:** Intensity ratios of the representative NIR bands $\left(R=\frac{I_{5907}}{I_{5564}}\right)$ of R-HDPE samples and PP fraction deduced by means of the calibration curve.

Sample	$R\;=\;\frac{I_{5907}}{I_{5564}}$	$C_{PP/PE}$ $\left(\frac{w}{w}\%\right)$
8	0.124	14.25
4	0.107	10.00
7	0.089	5.50
3	0.08	3.25
6	0.078	2.75
2	0.077	2.50
5	0.073	1.50
1	0.068	0.25

## Data Availability

The data presented in this study are available upon request from the corresponding author.

## Abbreviations

The following abbreviations are used in this manuscript:

HDPE	High-Density Polyethylene
R-HDPE	Recycled High-Density Polyethylene
LDPE	Low-Density Polyethylene
PP	Polypropylene (isotactic)
ATR	Attenuated Total Reflectance (spectroscopy)
NIR	Near-Infrared (spectroscopy)
